# Multifocal stimulation of the cerebro-cerebellar loop during the acquisition of a novel motor skill

**DOI:** 10.1038/s41598-021-81154-2

**Published:** 2021-01-19

**Authors:** Maximilian J. Wessel, Chang-hyun Park, Elena Beanato, Estelle A. Cuttaz, Jan E. Timmermann, Robert Schulz, Takuya Morishita, Philipp J. Koch, Friedhelm C. Hummel

**Affiliations:** 1 Defitech Chair of Clinical Neuroengineering, Center for Neuroprosthetics (CNP) and Brain Mind Institute (BMI), Swiss Federal Institute of Technology Lausanne (EPFL), Campus Biotech, Chemin des Mines 9, 1202 Geneva, Switzerland; 2 Defitech Chair of Clinical Neuroengineering, Clinique Romande de Réadaptation, Center for Neuroprosthetics (CNP) and Brain Mind Institute (BMI), Swiss Federal Institute of Technology (EPFL Valais), Sion, Switzerland; 3 Department of Neurology, University Medical Center Hamburg-Eppendorf, Hamburg, Germany; 4 Clinical Neuroscience, University of Geneva Medical School, Geneva, Switzerland

**Keywords:** Neuroscience, Cognitive neuroscience, Learning and memory

## Abstract

Transcranial direct current stimulation (tDCS)-based interventions for augmenting motor learning are gaining interest in systems neuroscience and clinical research. Current approaches focus largely on monofocal motorcortical stimulation. Innovative stimulation protocols, accounting for motor learning related brain network interactions also, may further enhance effect sizes. Here, we tested different stimulation approaches targeting the cerebro-cerebellar loop. Forty young, healthy participants trained a fine motor skill with concurrent tDCS in four sessions over two days, testing the following conditions: (1) monofocal motorcortical, (2) sham, (3) monofocal cerebellar, or (4) sequential multifocal motorcortico-cerebellar stimulation in a double-blind, parallel design. Skill retention was assessed after circa 10 and 20 days. Furthermore, potential underlying mechanisms were studied, applying paired-pulse transcranial magnetic stimulation and multimodal magnetic resonance imaging-based techniques. Multisession motorcortical stimulation facilitated skill acquisition, when compared with sham. The data failed to reveal beneficial effects of monofocal cerebellar or additive effects of sequential multifocal motorcortico-cerebellar stimulation. Multimodal multiple linear regression modelling identified baseline task performance and structural integrity of the bilateral superior cerebellar peduncle as the most influential predictors for training success. Multisession application of motorcortical tDCS in several daily sessions may further boost motor training efficiency. This has potential implications for future rehabilitation trials.

## Introduction

The acquisition of novel motor skills is a core ability of human behaviour. Examples emphasizing its importance are learning of new professional manual skills, sports, music, or the familiarization with novel technological devices. This great significance of motor skill learning raises the intriguing idea of designing novel technology-based interventions, which may further augment the learning success.

One of such approaches is the combination of behavioural training with transcranial direct current stimulation (tDCS). First proof-of-principle work investigating this approach, mainly targeting the primary motor cortex (M1), is promising^1,2^. However, several constraints have been identified—namely variable effect sizes between studies, considerable inter-individual variability, and limited mechanistic understanding^3^. These constraints call for further protocol optimizations. One approach is to design novel and innovative stimulation approaches accounting also for brain network interactions underlying motor control and learning. This strategy may further enhance neuromodulatory effects by mimicking natural aspects of neuronal network processing. Additionally, multisession application might lead to more homogenous and larger effect sizes.

In the present study, we strove to work on these challenges focusing on optimizing tDCS protocols targeting the cerebro-cerebellar loop accounting for its fundamental role in motor control, learning, and cognitive processing^4,5^. Based on the main hubs of the motor network involved in motor skill acquisition, i.e., M1 and the cerebellum (CB), we empirically tested different protocol optimization approaches for which we formulated specific research questions (RQs). RQ1 was: can multisession tDCS of M1 concurrently applied to a motor training lead to additive effects? M1 currently constitutes the core and by far the most frequently investigated target for promoting motor skill learning^3^ accounting for its well established functional role^6,7^ and relative good accessibility with non-invasive techniques. Theoretical considerations^8^ and meta-analyses^9^ have suggested that an increase of dosage may further strengthen effects. However, additional experimental validation is needed.

Secondly, we investigated in RQ2, if stimulation of alternative targets within the motor network, such as the CB, can enhance motor skill learning. This was based on the assumption that broadening of the spectrum of potential stimulation targets may enhance effect sizes at group level. We chose to further investigate the potential of cerebellar stimulation accounting for its high potential to undergo neuroplastic changes^10^, its broad connections and herby potential influence on various neocortical regions^11^, and the promising data from first proof-of-principle studies^12,13^.

Thirdly, we assessed in RQ3, if multifocal stimulation of the cerebro-cerebellar loop can further strengthen effects. Multifocal stimulation offers the advantage to mimic aspects of natural brain processing, such as joint neuronal operations in connected brain areas^14^ and achieve a synergistic effect based on the respective contributions to the learning process. Specifically, we chose to stimulate the motorcortico-cerebellar loop in two sessions per day on two consecutive days. M1 stimulation was applied in the first session and was succeeded by cerebellar stimulation in the second daily session to achieve additive, synergistic effects. This was based on our in-lab findings that M1 stimulation mainly exerts its effects via the facilitation of online learning and cerebellar stimulation via the promotion of offline learning effects^2,13^. We speculated that the multifocal stimulation approach might further boost tDCS-mediated learning enhancement via sequentially engaging complementary plasticity mechanism—long-term potentiation (LTP)-like process^2,15^ in M1 during the first daily session and optimally shaping of cerebellar cortex activity in the second daily session and the immediate post-training phase^13,16^. Thus, we acknowledge that there is certainly an ongoing debate in the field on the most susceptible temporal components and also task-specific effects might play a large role^3,17^.

Fourthly, we strove to investigate potential underlying mechanisms addressing RQ4: are GABAergic and glutamatergic neurotransmission in motorcortical circuits modulated by the combined application of tDCS and motor training? The potential modulation of neurotransmission was quantified with paired-pulse transcranial magnetic stimulation (ppTMS) techniques^18–21^.

Lastly, in RQ5 we investigated whether multimodal data consisting of behavioural, ppTMS and magnetic resonance imaging (MRI)-based parameters can predict the magnitude of training success and/or stimulation response. Previously, predictors based on systems neuroscience techniques have been associated with success of training-based interventions^22–26^. However, prior studies mainly applied approaches exploiting a single modality. We hypothesized that the combination of multimodal data in a combined predictive model may further improve model performance.

## Results

### RQ1: Additive effect of multisession monofocal M1 stimulation

**Figure 1:**
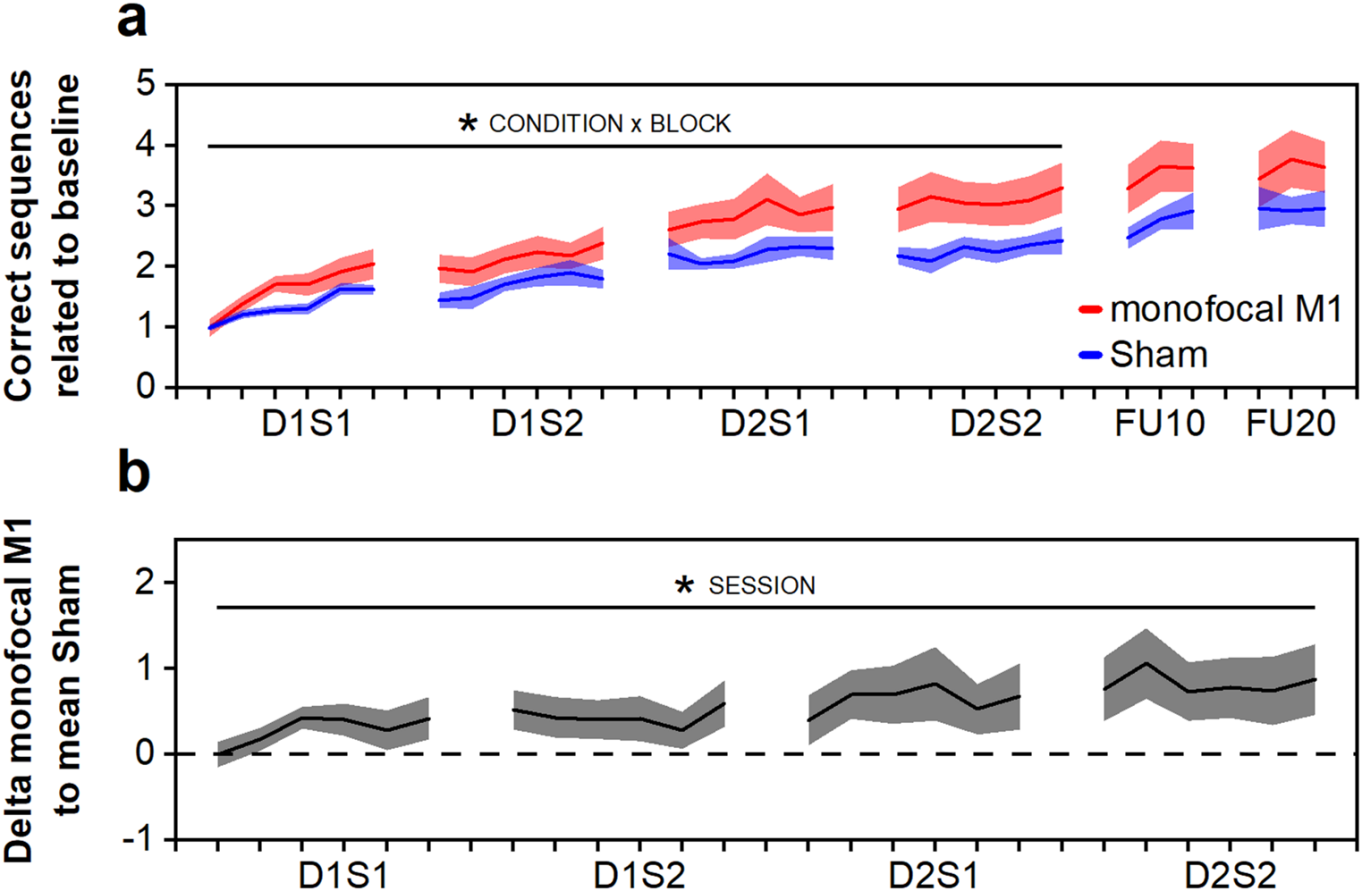
Multisession monofocal M1 stimulation. D1S1, day 1 session 1; D1S2, day 1 session 2; D2S1, day 2 session 1; D2S2, day 2 session 2; FU10, circa. 10 day follow-up; FU20, circa. 20 day follow-up. Margins of error correspond to standard error of the mean. (**a**) Learning curves of the monofocal M1 (red) and the sham group (blue). (**b**) Analysis of additive effects. Depicted is the mean of the individual learning trajectories of the monofocal M1 group related to the mean sham trajectory during the training phase. * depicts *p* < 0.05 for (**a**) interaction effect CONDITION × BLOCK, (**b**) main effect SESSION.

To address RQ1, learning data from the monofocal M1 group were compared with the sham group. The analysis of the training phase revealed a significant effect of CONDITION (χ^2^(1) = 3.91, *p* = 0.048), BLOCK (χ^2^(23) = 416.16, *p* < 0.001), and a CONDITION × BLOCK interaction (χ^2^(23) = 38.36, *p* = 0.023). In conjunction with the descriptive statistics these data indicate manifest skill learning in both groups and strengthened learning in the monofocal M1 group, when compared with sham. Please see also Fig. 1a. Additionally, motor training (SESSION: χ^2^(4) = 28.31, *p* < 0.001), but not stimulation (CONDITION: χ^2^(1) = 2.00, *p* = 0.16) enhanced simple task performance as measured via intermingled performance probes (blocks testing a pseudorandom, not prior trained motor sequence), see Supplementary Table S1.

To evaluate if successive sessions of monofocal M1 stimulation led to additive effects, we contrasted (subtraction) the individual learning trajectories of the monofocal M1 group with the mean trajectory of the sham group. The analysis indicated a significant SESSION effect (χ^2^(3) = 9.67, *p* = 0.022), suggesting additive effects of successive monofocal M1 stimulation sessions. This finding was further strengthened by a significant (*p* = 0.024) post hoc contrast comparing the last session on day 2 (D2S2) to the first session on day 1 (D1S1). Please see also Fig. 1b.

Analysis of the retention phase revealed a significant effect of SESSION (χ^2^(1) = 5.61, *p* = 0.018), but not of CONDITION (χ^2^(1) = 1.77, *p* = 0.18), or CONDITION × SESSION interaction (χ^2^(1) = 2.21, *p* = 0.14), pointing towards no stimulation-associated effects on task retention.

Emerging features in the analyses of temporal components were that online learning in D1S1 was significantly larger than in the other training sessions—contrasts to: D1S2 *p* = 0.041, D2S1 *p* = 0.0032, D2S2 *p* = 0.013. Furthermore, offline learning overnight was significantly larger than within day 1 (*p* = 0.036). Please see also Table 1 for full analysis of temporal components.

**Table 1:** Temporal components of learning.

Research question	M ± SEM (chronological order)	CONDITION	SESSION	CONDITION x SESSION	Post hocs
**(a) Online learning**					
RQ1 (M1 vs. Sham)	M1: 1.05 ± 0.27, 0.42 ± 0.15, 0.36 ± 0.30, 0.36 ± 0.19 Sham: 0.63 ± 0.08, 0.34 ± 0.20, 0.09 ± 0.11, 0.25 ± 0.18	χ^2^(1) = 1.56, *p* = 0.21	χ^2^(3) = 14.71, *p* = 0.0021*	χ^2^(3) = 1.41, *p* = 0.70	D1S1 versus D1S2: *p* = 0.041* D1S1 versus D2S1: *p* = 0.0032* D1S1 versus D2S2: *p* = 0.013* D1S2 versus D2S1: *p* = 0.80 D1S2 versus D2S2: *p* = 0.97 D2S1 versus D2S2: *p* = 0.96
RQ2 (CB vs. Sham)	CB: 0.50 ± 0.15, 0.29 ± 0.11, 0.26 ± 0.14, 0.37 ± 0.12 Sham: 0.63 ± 0.08, 0.34 ± 0.20, 0.09 ± 0.11, 0.25 ± 0.18	χ^2^(1) = 0.04, *p* = 0.83	χ^2^(3) = 9.52, *p* = 0.023*	χ^2^(3) = 2.06, *p* = 0.56	D1S1 versus D1S2: *p* = 0.22 D1S1 versus D2S1: *p* = 0.016* D1S1 versus D2S2: *p* = 0.21 D1S2 versus D2S1: *p* = 0.67 D1S2 versus D2S2: *p* = 1.00 D2S1 versus D2S2: *p* = 0.69
RQ3 (M1-CB vs. M1)	M1-CB: 0.76 ± 0.11, 0.45 ± 0.12, 0.42 ± 0.11, 0.24 ± 0.15 M1: 1.05 ± 0.27, 0.42 ± 0.15, 0.36 ± 0.30, 0.36 ± 0.19	χ^2^(1) = 0.24, *p* = 0.62	χ^2^(3) = 15.52, *p* = 0.0014*	χ^2^(3) = 1.56, *p* = 0.67	D1S1 versus D1S2: *p* = 0.024* D1S1 versus D2S1: *p* = 0.012* D1S1 versus D2S2: *p* = 0.0023* D1S2 versus D2S1: *p* = 0.99 D1S2 versus D2S2: *p* = 0.85 D2S1 versus D2S2: *p* = 0.95
**(b) Offline learning**					
RQ1 (M1 vs. Sham)	M1: − 0.08 ± 0.10, 0.23 ± 0.29, − 0.04 ± 0.16 Sham: − 0.17 ± 0.08, 0.42 ± 0.18, − 0.12 ± 0.18	χ^2^(1) < 0.01, *p* = 0.98	χ^2^(2) = 7.98, *p* = 0.018*	χ^2^(2) = 0.93, *p* = 0.63	D1_withinday_ versus D1D2_overnight_ *p* = 0.036* D1_withinday_ versus D2_withinday_ *p* = 0.96 D1D2_overnight_ versus D2_withinday_ *p* = 0.066
RQ2 (CB vs. Sham)	CB: 0.06 ± 0.12, 0.13 ± 0.09, − 0.21 ± 0.17 Sham: − 0.17 ± 0.08, 0.42 ± 0.18, − 0.12 ± 0.18	χ^2^(1) = 0.14, *p* = 0.71	χ^2^(2) = 10.13, *p* = 0.0063*	χ^2^(2) = 3.76, *p* = 0.15	D1_withinday_ versus D1D2_overnight_ *p* = 0.064 D1_withinday_ versus D2_withinday_ *p* = 0.73 D1D2_overnight_ versus D2_withinday_ *p* = 0.0098*
RQ3 (M1-CB vs. M1)	M1-CB: − 0.05 ± 0.17, 0.15 ± 0.13, − 0.38 ± 0.06 M1: − 0.08 ± 0.10, 0.23 ± 0.29, − 0.04 ± 0.16	χ^2^(1) = 0.91, *p* = 0.34	χ^2^(2) = 5.95, *p* = 0.051	χ^2^(2) = 1.37, *p* = 0.50	n/a

(a) Analysis of online learning, operationalised as difference between the last and the first block of a given session. (b) Analysis of offline learning defined as difference between the first block of the subsequent session and the last block of the preceding session. M1, monofocal M1 stimulation group; Sham, sham group; CB, monofocal cerebellar stimulation group; M1-CB, multifocal motorcortical-cerebellar stimulation group; M, mean; SEM, standard error of the mean. * depicts *p* < 0.05.

### RQ2: Multisession cerebellar stimulation

**Figure 2:**
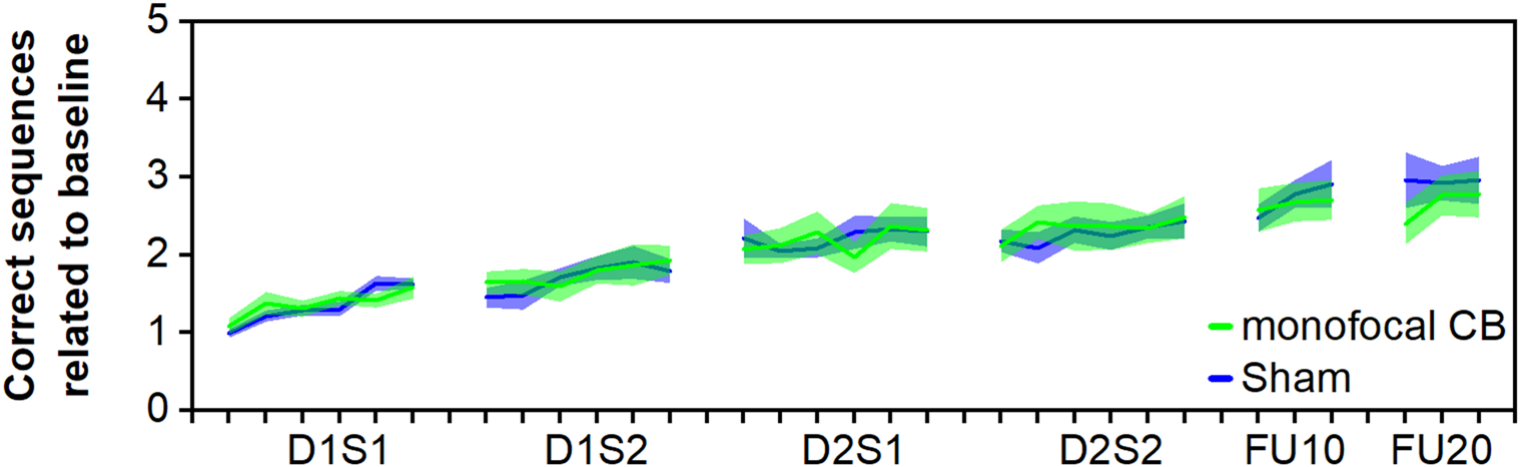
Multisession monofocal CB stimulation. D1S1, day 1 session 1; D1S2, day 1 session 2; D2S1, day 2 session 1; D2S2, day 2 session 2; FU10, circa 10 day follow-up; FU20, circa 20 day follow-up. Margins of error correspond to standard error of the mean.

We chose the left CB as target of interest with the aim to modulate cerebellar representations of the ipsilateral (left) training hand. The learning trajectory of the monofocal CB group was compared with the sham group. Statistical analysis revealed a significant effect of BLOCK (χ^2^(23) = 374.52, *p* < 0.001), but not of CONDITION (χ^2^(1) = 0.03, *p* = 0.86) or CONDITION × BLOCK interaction (χ^2^(23) = 18.19, *p* = 0.75), demonstrating manifest skill learning, but no stimulation-associated effects on the training phase. Furthermore, motor training (SESSION: χ^2^(4) = 19.58, *p* < 0.001), but not stimulation (CONDITION: χ^2^(1) = 0.01, *p* = 0.92) had a positive effect on simple task performance, see Supplementary Table S1. The analysis of the retention phase indicated a trend for SESSION (χ^2^(1) = 2.76, *p* = 0.097). No significant effects for CONDITION (χ^2^(1) = 1.20, *p* = 0.27), or a CONDITION × SESSION interaction (χ^2^(1) = 1.98, *p* = 0.16) were present, pointing towards no stimulation-associated effects on task retention. Please see also Fig. 2.

Analysis of temporal components of learning revealed a significantly larger online learning in D1S1 than in the first session on day 2(D2S1) (*p* = 0.016). Moreover, overnight offline learning was significantly larger than offline learning between the sessions on day 2 (*p* = 0.0098), see also Table 1.

### RQ3: Sequential multifocal motorcortico-cerebellar stimulation

**Figure 3:**
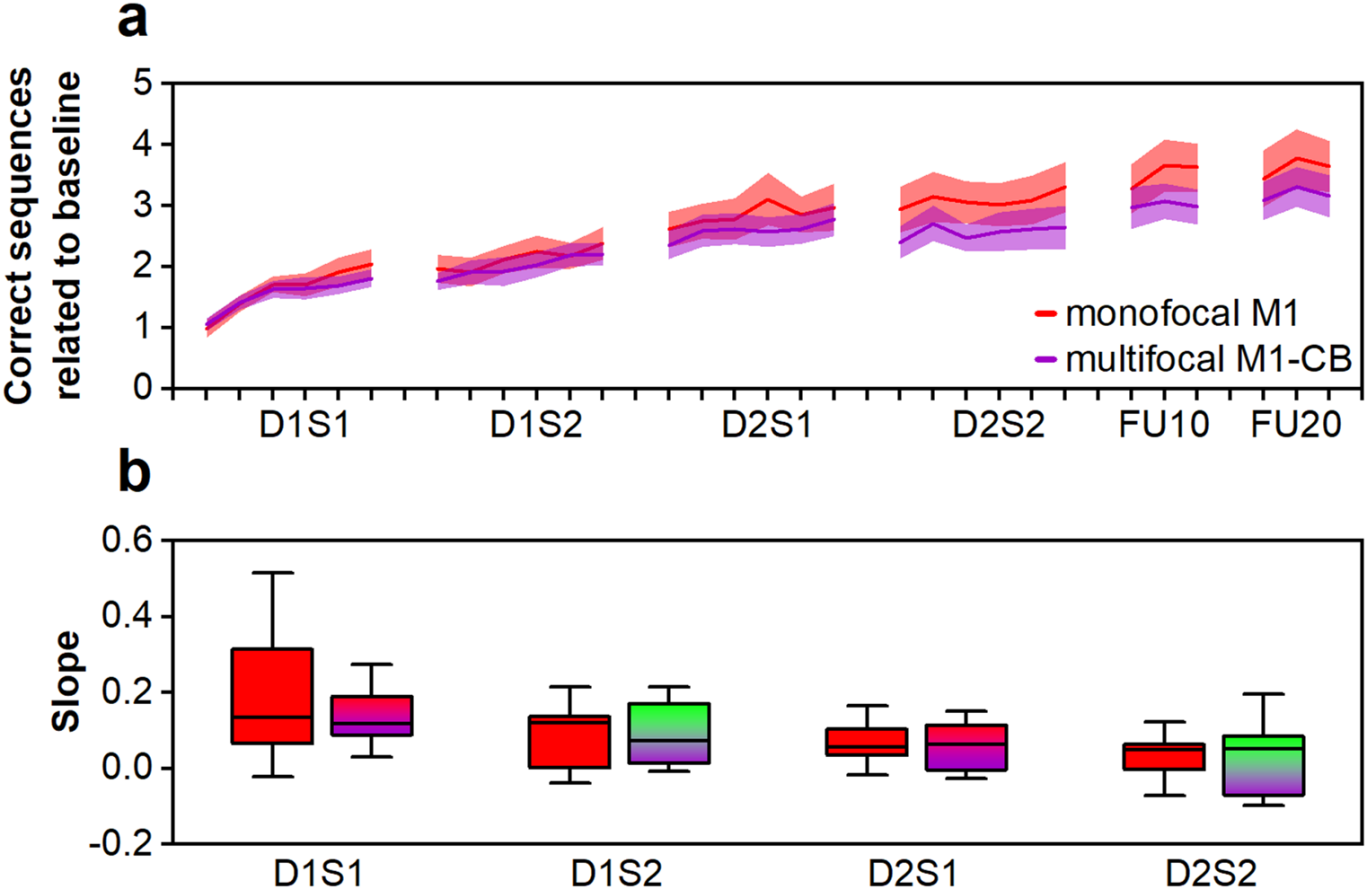
Sequential multifocal M1-CB stimulation. D1S1, day 1 session 1; D1S2, day 1 session 2; D2S1, day 2 session 1; D2S2, day 2 session 2; FU10, ca. 10 day follow-up; FU20, ca. 20 day follow-up. (**a**) Learning curves of the monofocal M1 (red) and the multifocal M1-CB group (violet). Margins of error correspond to standard error of the mean. (**b**) Comparison of slope of the learning trajectories during the training phase ordered per SESSION and CONDITION. Monofocal M1 group (red), multifocal M1-CB group at a session in which M1 stimulation was applied (gradient fill red-violet), multifocal M1-CB stimulation group at a session in which CB stimulation was applied (gradient fill green-violet). Box plots depict median (solid vertical line), box bounds (upper to lower quartile), whisker (range within 1.5 interquartile range).

To assess for potential beneficial additive effects of sequential multifocal stimulation, we compared the multifocal M1-CB group with the monofocal M1 group. The analysis demonstrated a significant effect of BLOCK (χ^2^(23) = 439.89, *p* < 0.001), but not of CONDITION (χ^2^(1) = 0.65, *p* = 0.42) or a CONDITION × BLOCK interaction (χ^2^(23) = 26.27, *p* = 0.29), and hereby was not supportive of the hypothesis of additive effects of multifocal stimulation. Please see also Fig. 3a. Subsequently, we further investigated, if participants in the multifocal M1-CB group presented reduced learning in sessions in which they received cerebellar stimulation (D1S2 and D2S2) by comparing the slope of the learning curves in the delimited training sessions with the monofocal M1 group, please see Fig. 3b. Statistical analysis revealed no significant CONDITION × SESSION interaction (χ^2^(3) = 1.60, *p* = 0.66), thus rejecting this hypothesis. Additionally, motor training (SESSION: χ^2^(4) = 46.37, *p* < 0.001), but not stimulation (CONDITION: χ^2^(1) = 1.84, *p* = 0.18) enhanced simple task performance, for post hoc testing see Supplementary Table S1.

Moreover, analysis of the retention phase revealed no significant effects of CONDITION (χ^2^(1) = 1.16, *p* = 0.28), SESSION (χ^2^(1) = 2.66, *p* = 0.10), or CONDITION × SESSION interaction (χ^2^(1) = 0.61, *p* = 0.43), pointing towards no stimulation-associated effects on task retention.

Analysis of temporal components identified greater online learning in D1S1 in comparison with the other sessions as significant apparent feature—contrast to: D1S2 *p* = 0.024, D2S1 *p* = 0.012, D2S2 *p* = 0.0023. Please see also Table 1.

### RQ4: Analysis of intracortical inhibition and facilitation

**Figure 4:**
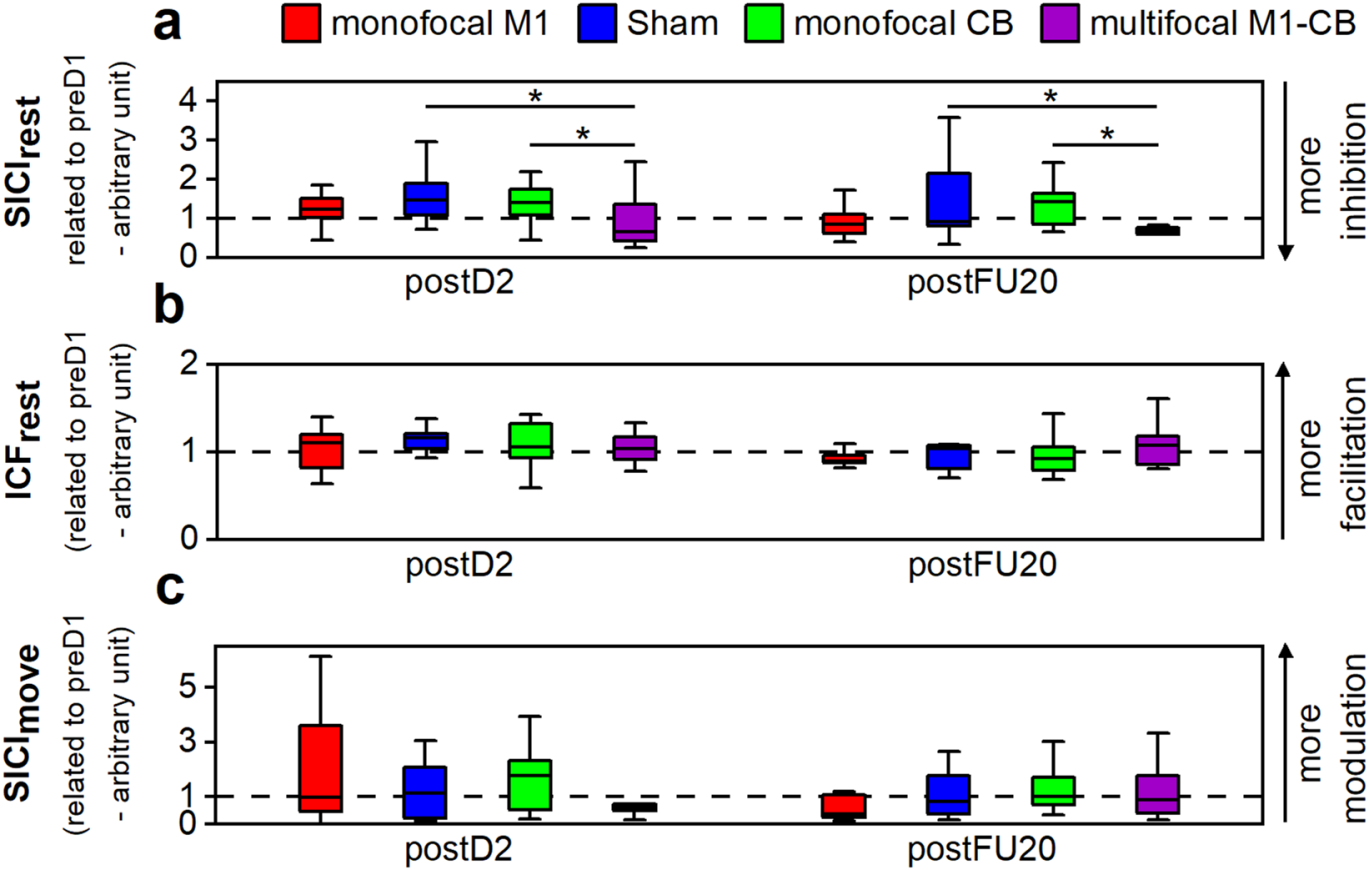
Paired-pulse TMS assessments. postD2, after training day 2; postFU20, after follow-up day 20. Red, monofocal M1 group; blue, sham group; green, monofocal CB group; violet, multifocal M1-CB group. Box plots depict median (solid vertical line), box bounds (upper to lower quartile), whisker (range within 1.5 interquartile range). Values are related to baseline by computing the ratio between postD2 and preD1, respectively postFU20 and preD1. (**a**) SICI_rest_ related to baseline (preD1), * depicts *p* < 0.05 for post hoc mean-separation testing across significant main effect CONDITION applying a Tukey adjustment. The SICI_rest_ data was log-transformed for statistical analysis to meet the normality of residuals assumption. (**b**) ICF_rest_ related to baseline (preD1). (**c**) SICI_move_ modulation, expressed as absolute value of the delta between 90 and 20% of reaction time (RT) datapoints, related to baseline (preD1).

The analysis of the resting motor threshold (RMT) did not suggest differences in RMT across conditions or changes over time (CONDITION: χ^2^(3) = 2.66, *p* = 0.45; SESSION: χ^2^(2) = 2.33, *p* = 0.31; CONDITION × SESSION: χ^2^(6) = 2.53, *p* = 0.87). The analysis of the modulation of short intracortical inhibition assessed at rest (SICI_rest_) compared to baseline (preD1) revealed a significant effect of CONDITION (χ^2^(3) = 13.63, *p* = 0.0035) and a trend for SESSION (χ^2^(1) = 3.50, *p* = 0.061), with a non-significant CONDITION × SESSION interaction (χ^2^(3) = 2.37, *p* = 0.50), please see also Fig. 4a. Post hoc testing indicated a significant group difference for the multifocal M1-CB to sham (*p* = 0.0078) and the multifocal M1-CB to monofocal CB (*p* = 0.013) contrasts, suggesting pronounced inhibition throughout the course of learning in the multifocal stimulation group. Auxiliary analysis did not reveal a significant modulation from baseline for all assessed time points (one sample t-test, Bonferroni-corrected: for all comparisons *p* > 0.05). Spearman's rank correlations revealed no significant associations between training gain (whole group: r_s_ = 0.045, *p* = 0.79; multifocal-only: r_s_ = 0.52, *p* = 0.16) or retention at FU20 (whole group: r_s_ = 0.19, *p* = 0.26; multifocal-only: r_s_ = 0.17, *p* = 0.68) with the modulation of SICI_rest_.

The analysis of the modulation of intracortical facilitation at rest (ICF_rest_) compared to baseline indicated a strong trend for SESSION (χ^2^(1) = 3.81, *p* = 0.051), pointing towards a reduction of facilitation after the follow-up session. There was no significant effect of CONDITION (χ^2^(3) = 2.02, *p* = 0.57) or of a CONDITION × SESSION interaction (χ^2^(3) = 5.47, *p* = 0.14), please see Fig. 4b. Auxiliary analysis did not reveal a significant modulation from baseline for all assessed time points (one sample t-test, Bonferroni-corrected: for all comparisons *p* > 0.05). Spearman's rank correlations revealed no significant associations between training gain (r_s_ = 0.19 *p* = 0.26) or retention at FU20 (r_s_ = 0.18, *p* = 0.28) with the modulation of ICF_rest_.

Finally, analysis of the event-related SICI_move_ modulation compared to baseline indicated no significant effect on CONDITION (χ^2^(3) = 1.00, *p* = 0.80). There was a trend for SESSION (χ^2^(1) = 3.55, *p* = 0.060) providing a slight indication for more modulation in postD2. Moreover, the analysis revealed no significant CONDITION × SESSION interaction (χ^2^(3) = 4.63, *p* = 0.20). Auxiliary analysis did not reveal a significant modulation from baseline for all assessed time points (one sample t-test, Bonferroni-corrected: for all comparisons *p* > 0.05). Please see Fig. 4c. Spearman's rank correlations indicated no significant association between training gain (r_s_ = − 0.26, *p* = 0.12) or retention at FU20 (r_s_ = − 0.13, *p* = 0.45) with the modulation of SICI_move_.

### RQ5: Prediction of training gain

**Figure 5:**
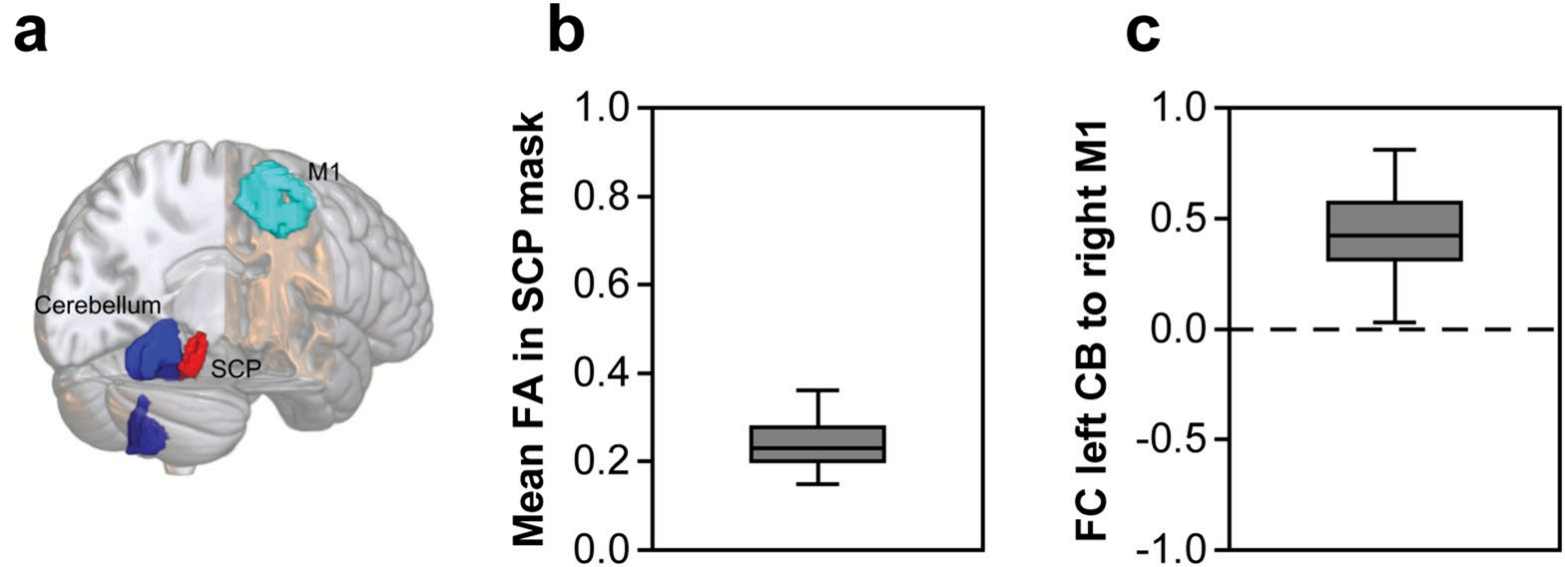
(**a**) Overview of the regions of interest (ROIs) for MRI analysis visualized with MRIcroGL (https://www.mccauslandcenter.sc.edu/mricrogl/). For rsMRI: blue, motor part of the left cerebellum (CB); light blue, right primary motor cortex (M1). For DWI: red, bilateral superior cerebellar peduncle (SCP). (**b**) Mean FA in the SCP mask, (**c**) Functional connectivity (FC) between left CB and right M1. Box plots depict median (solid vertical line), box bounds (upper to lower quartile), whisker (range within 1.5 interquartile range).

**Figure 6:**
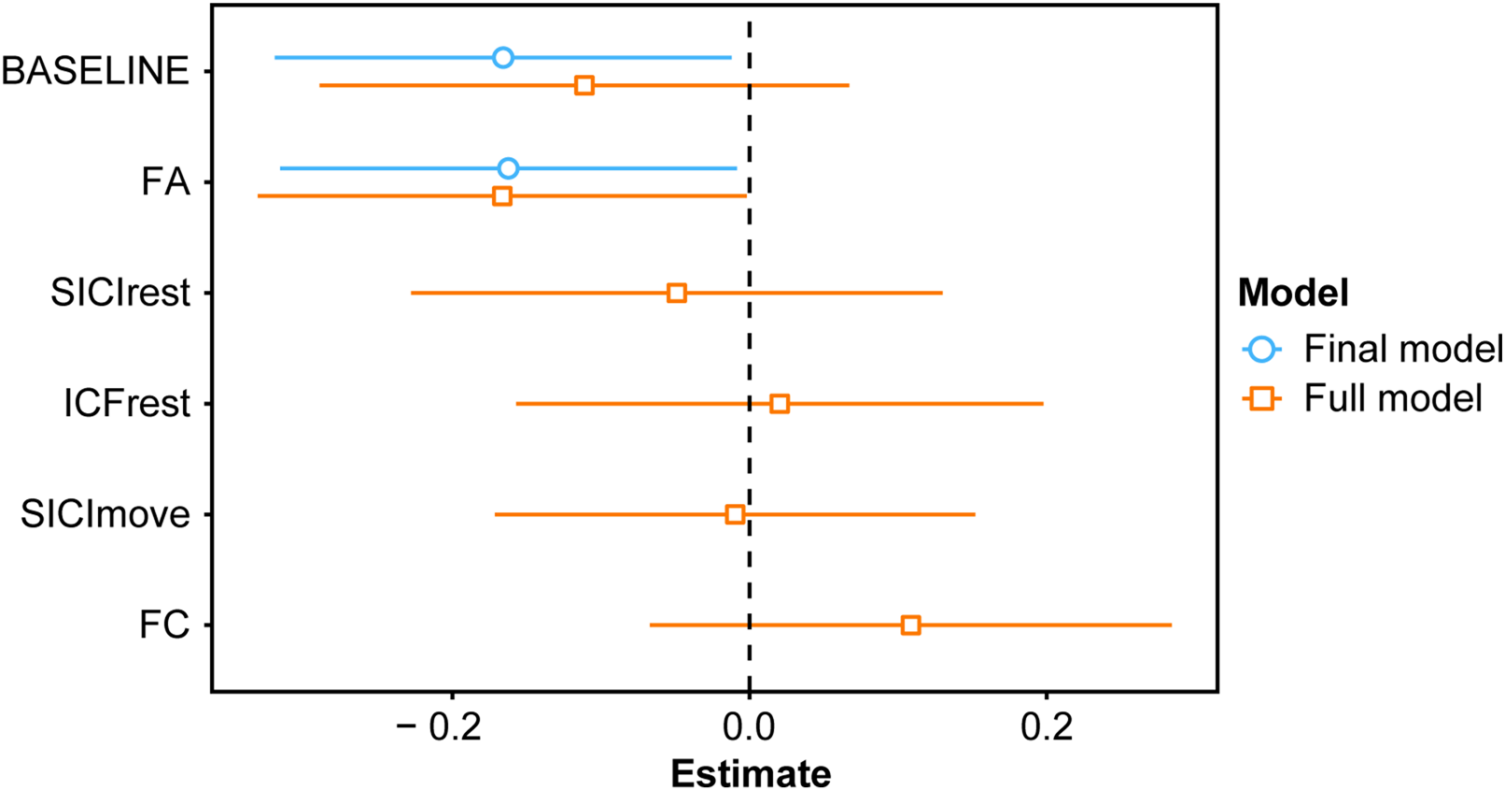
Effect plot depicts weights of beta coefficients (estimates) for full (orange) and final (blue) multiple linear regression model predicting training gain. Assessed predictors: (1) BASELINE—performance in the SFTT at baseline, (2) SICI_rest_ at preD1, (3) ICF_rest_ at preD1, (4) SICI_move_ modulation at preD1, (5) mean FA within the SCP mask, (6) FC between the left cerebellum and right M1. Final model was obtained by stepwise backward selection based on the Akaike information criterion (AIC). Margins of error depict 95% confidence intervals.

In addition to behavioural performance parameters and ppTMS surrogates of GABAergic and glutamatergic neurotransmission^18^, we assessed the potential of MRI-derived parameters to predict training gain. Specifically, (1) we computed mean fractional anisotropy (FA) of the bilateral superior cerebellar peduncle (SCP) with diffusion-weighted MRI to characterize the microstructural integrity of the cerebellothalamic fibres^27^. Furthermore, based on prior research linking resting-state functional connectivity (FC) in the cerebello-cortical loop with the gain in motor sequence learning^28^, we computed (2) region of interest (ROI) to ROI based FC between the left cerebellum and the right M1 employing the resting-state fMRI (rs-fMRI) data, for an overview of the selected ROIs please see Fig. 5a. The results are depicted in Fig. 5b,c. Subsequently, we applied a stepwise multiple linear regression analysis to find the best fitting model to predict training gain by applying a backward selection procedure (for further details please see methods section below). The full model included the following predictors: (1) BASELINE—performance in the sequential finger tapping task (SFTT) at baseline, (2) SICI_rest_ at preD1, (3) ICF_rest_ at preD1, (4) SICI_move_ modulation at preD1, (5) mean FA within the SCP mask, (6) FC between the left cerebellum and right M1. The final model was significant (F(2,37) = 4.08, *p* = 0.025), but explained only a limited proportion of variance (adjusted R^2^ = 0.14). Remaining and significant predictors were BASELINE (*p* = 0.035) and FA (*p* = 0.039). For the effect plot see Fig. 6.

Subsequently, we assessed with the same statistical approach, if it was also possible to predict responsiveness towards monofocal M1 tDCS by contrasting the individual training gain of the M1 group with the mean training gain of the sham group. The approach failed to predict this surrogate of responsiveness towards M1 tDCS (stepAIC approach identified the intercept only model as winning model), thus did not enable us to further differentiate responders from non-responders to M1 stimulation.

## Discussion

The main findings of the current study were: (1) that anodal tDCS applied to M1 enhances the acquisition of a novel motor skill and its repeated application results in additive effects, (2) the study failed to reveal any stimulation-associated effects on motor skill learning by monofocal cerebellar stimulation, (3) multifocal stimulation of the cerebro-cerebellar loop did not lead to additional additive effects, when studying a cohort of young healthy participants.

Our finding of beneficial effects of monofocal M1 stimulation (RQ1) extends the available literature investigating the potential of tDCS-based interventions to augment motor learning, for review please see Buch and colleagues^3^. However, the robustness of the approach has been recently called into question^3,9^. One matter of debate are the potential underlying mechanisms of action, such as the susceptible temporal components of learning. In this regard, Reis and colleagues indicated in their seminal work that the beneficial effect of anodal M1 tDCS was mainly mediated by an enhancement of offline effects^1^, when studying a sequential visual isometric pinch task. As a potential underlying mechanism the authors propose a delayed enhancement of learning-related protein synthesis rather than immediate LTP-like effects^1^. Our data failed to reveal stimulation-associated effects on decomposable temporal components of learning, when studying a different learning task (SFTT). This discrepancy might be explained by task-specific effects^17^. We speculate that probably both discussed mechanisms have been engaged in mediating the tDCS-related effects in the present experiment.

A further challenge of the field is to develop novel strategies, which might improve protocol robustness. In this regard, the present study design extends prior work by testing a twice daily application architecture. The results indicate a steady increase in group difference up to the last training session pointing towards potential additive effects of the repeated tDCS applications. We speculate that these additive effects were potentially mediated by conjointly engaging fast and slow learning processes and the respective underlying brain plasticity^29^. It is of note that the optimal timing of protocol application might be of crucial importance. Our rational to choose a 90 min inter-session-interval for the within-day sessions was to stimulate in a phase of still anticipated enduring effects^30^, but avoiding too short inter-session-intervals, which have been linked to unfavourable homeostatic interactions^31^. Further systematic investigations on optimal session architecture of spaced application protocols^32^ in combination with motor learning constitute a promising direction for future research.

In a second optimization approach (RQ2), we evaluated potential effects of modulating a different key area of the motor learning network by means of monofocal cerebellar stimulation. Our data failed to reveal stimulation-associated effects for anodal cerebellar tDCS studying the SFTT. This is in contrast to prior proof-of-principle work indicating positive effects on motor learning, when studying visuomotor adaptation^33^, implicit motor learning^34^, or motor skill learning^12,13^. Several reasons may explain the null results. At first, the SFTT seems to be less dependent on cerebellar resources as classical cerebellum-dependent learning tasks, as for instances motor adaptation paradigms. The learning and organization of novel sequential finger movements relies also on other brain structures, such as M1^7^, the striatum^35^, or the supplementary motor area^36^. In addition to cerebellum-dependent sensory prediction error-based learning, other learning strategies are likely of importance for successful skill acquisition in the SFTT. Moreover, recent follow-up studies have raised questions on the reliability of cerebellar tDCS protocols to enhance motor learning^37,38^. Discussed reasons for the limited robustness of monofocal cerebellar tDCS protocols are the high susceptibility towards variations in task parameters^37^, the large inter-individual differences in the lobule-specific distribution of the applied electric field^39^, and the individual brain-derived neurotrophic factor genotype^40^. One future approach to potentially overcome this current limitation of monofocal cerebellar tDCS might be protocol personalization based on computational modelling approaches, such as electric field dosimetry^41^. Importantly, alternative non-invasive brain stimulation techniques, such as cerebellar Theta Burst Stimulation, has shown to enhance visuo-motor adaptation in healthy subjects^42^ and beneficial effects on gait and balance functions in chronic stroke survivors^43^, and should be considered as an alternative cerebellar neuromodulation strategy.

In a third optimization approach (RQ3), we tested a multifocal motorcortico-cerebellar stimulation protocol applied concurrently to the motor training. Our rational to test the daily application sequence of M1 stimulation followed by cerebellar stimulation spaced by a 90 min inter-session interval was based on our recent data indicating enhancement of mainly online learning components by M1 tDCS^2,24^ and offline components by cerebellar tDCS^13^. The 90 min inter-session-interval was chosen to apply the cerebellar stimulation in a time window of anticipated enduring M1-stimulation-induced effects and for avoiding potentially interfering homeostatic interactions (as discussed above). We did not choose a simultaneous dual-site stimulation approach as at the time point of study implementation, the effects of electric field interferences by concurrent conventional M1 and cerebellar tDCS were unpredictable. The present data failed to reveal an additional benefit of multifocal stimulation of the cerebro-cerebellar loop, when compared with monofocal M1 stimulation, on learning a novel motor skill based on the sequential execution of fine finger movements in a cohort of young healthy participants. Visual inspection indicated that in fact the largest difference in variability between groups occurred in the first training session on day one, in which both interventional groups received comparable anodal M1 tDCS. However, response to multifocal M1-CB stimulation protocols might be different in conditions with pathological disbalanced cerebro-cerebellar interactions, such as after a stroke^44^. A further approach could be to target the cerebro-cerebellar loop in an inverted order of stimulation targets—CB first and contralateral M1 second. This approach could be particularly promising when studying adaptation to a novel visuomotor transformation, for which CB stimulation has shown to enhanced movement error reduction during the adaptation phase and M1 stimulation has shown to increase retention of the newly learnt visuomotor transformation^33^. This opposite functional dissociation in comparison to our prior work studying the acquisition of novel sequential finger movements^2,13^ points towards the importance of considering task-specific effects. The discussed alternative cerebello-cerebral tDCS protocol has been tested in first proof-of-principle work and has shown beneficial effects on upper limb tremor, hypermetria, and long-latency stretch reflexes in patients with cerebellar ataxia^45,46^. Another possibility would be to further implement simultaneous multifocal tDCS stimulation approaches, as tested in first studies recruiting patients with psychiatric disorders^47,48^. Both alternative stimulation approaches were outside the scope of our current research work, however should be further addressed in future.

The analyses of non-stimulation-associated features of learning revealed that motor sequence learning transferred to improved motor performance, when tested via an untrained motor sequence. This points towards a partial generalization of the acquired motor memory trace. Secondly, largest online learning occurred in the first session on day one, which may be explained by a partial saturation of LTP-like and ceiling effects. Lastly, overnight offline learning, when compared with within day offline learning tended to be of larger magnitude. However, the present study design does not allow to disentangle, if this was due to sleep-dependent consolidation effects^49^ or to simple passage of longer time (circa 90 min versus 24 h).

Regarding the ppTMS assessments, the data suggested a pronounced SICI_rest_ (GABA_A_-ergic) after task performance in the training and follow-up sessions in the multifocal stimulation group. At first sight, this seems to be unexpected, when considering previous findings. Based on the seminal work from Galea and colleagues^50^, the conventional view is that anodal cerebellar tDCS increases cerebello-brain inhibition (CBI) and stronger CBI has been linked to reduced SICI potentially via a reduced thalamocortical facilitation of inhibitory interneurons in M1^51^. We speculate that the preceding anodal M1 tDCS session, based on prior work^52^, might have reduced SICI earlier in the daily course of the experiment and hereby may have primed a greater susceptibility for inducing inhibitory net effects via homeostatic-like interactions^53^ in the successive training session. This pattern of inhibitory balance might have been re-established by subsequent task performance at the follow-up. It is of note that in well functioning young participants this slight change in inhibitory net balance was not associated with measurable behavioural consequences. For ICF_rest_, the data pointed towards a reduced facilitation after task performance at the follow-up sessions. This finding might be explained by a saturation of glutamatergic plasticity mechanisms in an advanced learning stage^54,55^. The analysis of SICI_move_ modulation suggested a trend for more modulation of GABA_A_-ergic circuits during movement preparation in the immediate post training phase, when compared with the evaluation after the follow-up sessions. This might be interpreted as a state of increased plasticity in GABA_A_-ergic circuits shortly after the motor training^56,57^. Visual inspection suggests that this tendency seemed pronounced in the M1 tDCS group showing a noteworthy dispersion of data at postD2. However, no clear association with behaviour (training gain) was present. It is important to note, that when applying other tasks and stimulation paradigms no effect of training phase^58^ or opposite tendencies^59^ on SICI_move_ modulation have been reported. In future work, it would be interesting to also study additional aspects of neurotransmission with TMS-based techniques for instance assessing long-interval intercortical inhibition (LICI—GABA_B_-ergic) or short-latency afferent inhibition (SAI—acetylcholinergic) to disentangle potential other underlying mechanisms of tDCS and motor learning^18^.

Lastly, multiple linear regression modelling identified baseline task performance and mean FA in the bilateral SCP as most influential predictors for the dependent variable training gain, with the final model explaining a limited proportion of variance (circa 14%). Baseline performance was negatively associated with the training gain, indicating that lower baseline performance was related to larger improvements in skill during the training phase. This could be explained by an increased exploratory behaviour in participants with lower baseline performance. Indeed, previous research have linked the amount of motor variability with training success^23^. To further substantiate this argument, we performed an exploratory analysis comparing the evolution of the coefficient of variance (CV) in the four training sessions for the primary outcome, number of correctly performed sequences, splitting the participants in a low and high baseline performer group via a median split. The analysis revealed a group difference (GROUP: χ^2^(1) = 17.10, *p* < 0.001) with a higher CV in the low performer group supporting the above discussed argument. An alternative explanation of the negative association between baseline and training gain are potentially emerging ceiling effects in the good baseline performer group. However, the continued tendency to improve at the follow-up sessions argues against this explanation.

Secondly, mean FA in the bilateral SCP was negatively associated with training gain. One possible explanation for this finding is that a pronounced cerebello-cortical output tract, constitutes a structural correlate of relatively exaggerated error processing, within the natural variability of a cohort of well-functioning healthy individuals. Yet, this interpretation remains highly speculative. It is of note, that conversely to the argument above higher FA values in the white matter adjacent to the dentate nuclei have been positively associated with the magnitude of motor skill learning in earlier work^60^. Overall, the assessed multimodal regression modelling approach indicated some potential for predicting motor training success and may complement available unimodal approaches^61,62^.

There are some limitations of the current work worthwhile to discuss. Conventional tDCS protocols lack spatial focality, which may have led to stimulation of adjacent, non-target brain areas. However, the functional consequences might be mitigated by concurrent task application, as task performance is assumed to partially channel the activation towards functionally relevant brain circuits. Secondly, our study failed to achieve the desired level of blinding. At whole group level, the participants guessed the nature of the applied tDCS (active versus sham) better than random chance (exact binomial test, *p* < 0.05). However, the sham group identified the correct stimulation type at chance level (exact binomial test, *p* = 1.00). Thirdly, the sample size of the current study is rather small, however by obtaining a significant result in a small sample suggests that the reported intervention effect is of a greater magnitude than an equivalent result obtained from studying a larger sample^63^. Conversely, the resulting low power may have hindered us for detecting small differences in-between groups. To inform future replication studies testing the effect of the here studied tDCS protocols on motor training, we now simulated power curves for increasing the sample size, see Supplementary Information and Supplementary Fig. S1. For RQ2 and 3, for which the applied tDCS protocols did not indicated a significant stimulation (CONDITION) effect in the current study, the simulation indicated that a sample size increase to N = 100 would not critically increase the level of power. Fourthly, as discussed above, alternative multifocal stimulation strategies of the cerebro-cerebellar loop, either sequentially stimulating first CB and then the contralateral M1 or simultaneous application strategies are promising, however were outside the scope of the current research work, and should be addressed in future.

To conclude, the present study contributes to the available literature indicating the potential of anodal M1 tDCS to enhance motor skill learning and further suggests a benefit of a twice daily application protocol. The data failed to reveal stimulation-associated effects of monofocal cerebellar tDCS or an additive effect of multifocal cerebro-cerebellar tDCS application, when studying a sample of well-functioning, young, healthy participants. However, both approaches should be tested and may have potential in conditions with disbalanced cerebro-cerebellar interactions, such as after a stroke^44^.

## Methods

### Participants

Forty young, healthy, right handed participants were recruited for the study (age 25.93 ± 3.47, 23 female). All participants were screened for and did not have any contraindications for non-invasive brain stimulation. The study was conducted in accordance to the Declaration of Helsinki^64^. All participants gave their written informed consent. The study protocol was approved by the local ethics committee of the Medical Association of Hamburg (PV3777). In the present study, we studied electrophysiological mechanisms in a cohort of young healthy study participants and not a healthcare-related intervention, for that reason the study was not conducted in the format of or registered as a clinical trial.

### Experimental design

**Figure 7:**
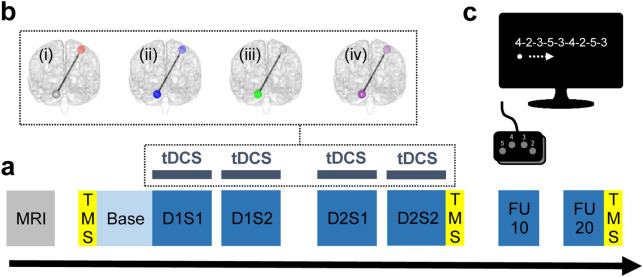
Experimental design. (**a**) Experimental timeline: a novel motor skill was trained over four sessions equally distributed over two training days (D1S1, D1S2, D2S1, D2S2). Prior to start of the training phase, baseline level of task performance was quantified (Base). Task retention was assessed circa 10 and circa 20 days after the training phase (FU10 and FU20). tDCS was applied simultaneously to the training sessions in a randomized, double-blind, sham controlled, parallel design applying one of the four conditions (please see also figure panel b): (1) red: monofocal M1 stimulation, (2) blue: sham stimulation, (3) green: monofocal cerebellar stimulation, (4) violet: sequential multifocal motorcortical-cerebellar stimulation (schematic illustrations created with BrainNet Viewer^85^). Further assessments included ppTMS (short intracortical inhibition—SICI at rest and during movement preparation, intracortical facilitation—ICF at rest) and multimodal MRI (T1-weighted anatomical, diffusion-weighted, rs-fMRI gradient-echo EPI images). (**c**) As a motor learning task, participants executed a modified version of the sequential finger tapping task (SFTT)^65,66^ with their non-dominant, left hand.

The participants carried out a motor training (for details on the specific task see below) on two daily 20 min sessions on two consecutive days. On each training day, the sessions were separated by a circa 90 min break. Baseline performance was assessed in a baseline block (Base) on day 1 prior to the start of the training phase. Task retention was assessed circa 10 and 20 days after the training phase (FU10 and FU20). TDCS was applied in a double-blind, sham-controlled, parallel design simultaneously to the training sessions. The motor learning sessions were embedded in ppTMS-based assessments before motor training on day 1 (preD1), after motor training on day 2 (postD2), and after the last follow-up session (postFU20). Furthermore, the participants were characterized with multimodal MRI-based neuroimaging before the start of the motor learning protocol or after its completion, in the second case respecting a wash-out period of at least 10 days after FU20 (median: 24 days, min: 10 days, max: 108 days). Please see also Fig. 7a for a depiction of the timeline.

### Motor learning task

The participants trained a modified version of the sequential finger tapping task^65,66^ (SFTT) with their non-dominant, left hand, please see also Fig. 7c. The training sessions lasted circa 20 min consisting of seven 90 s blocks (including the intermingled performance probe block, see below) separated by breaks. The follow-up sessions consisted of three blocks of 90 s also separated by breaks. The instruction was to repeatedly execute a nine elements motor sequence as rapidly and as accurately as possible on a four button keyboard, with each key 2 to 5 assigned to one finger, index (2) to little finger (5). To reduce the working memory load, the target sequence was displayed on a screen placed in front of the participants. A dot displayed below the chain of numbers served as a bookmark of current sequence position, but no feedback on task performance was provided. Different, but complexity-matched (Kolmogorov Complexity^67^), sequences were applied for baseline and the intermingled blocks serving as performance probes, for further details on the applied sequences please see Supplementary Table S2. The conduction of the SFTT was implemented in Presentation software (Neurobehavioral Systems Inc, Berkely, CA, United States).

### Transcranial direct current stimulation (tDCS)

Anodal tDCS was applied via a DC-stimulator (neuroConn, Ilmenau, Germany) using 5 × 5 cm sponge covered, conductive rubber, square electrodes soaked in saline solution. The stimulation was applied in a sham-controlled, double-blind, parallel design administering one of the four following pseudorandomly assigned conditions (please see also Fig. 7b): (1) monofocal M1 stimulation—current [I] = 1 mA, duration [T] = 20 min, fade-in/-out interval [Fi/o] = 8 s, montage: active electrode [E1] = TMS-based right motor hotspot^68^, return electrode [E2] = over contralateral supraorbital region^68^ (2) sham stimulation—T = 30 s other parameters were set to the M1 or cerebellar configuration based on a pseudorandom order, (3) monofocal cerebellar stimulation—I = 2 mA, T = 20 min, Fi/o = 8 s, E1 = 3 cm lateral to the inion^50^ over the left cerebellar hemisphere, E2 = over ipsilateral buccinators muscle^50^, (4) sequential multifocal motorcortical—cerebellar stimulation—active M1 or cerebellar stimulation (see above) applied in the following order, M1 stimulation during the first daily session and cerebellar stimulation during the second daily session. The electric field distribution of the above described cerebellar electrode montage has been recently re-evaluated applying finite element modeling analysis^39^. The analysis suggested that the applied electric field mainly affects lobules Crus I/II, VIIb, VIII, and IX^39^, and hereby reaches areas crucially involved in motor control located in the posterior cerebellar lobe (lobule VIII)^69^. The site-specific dose adjustment—1 mA for M1 and 2 mA for CB—was chosen based on our prior work documenting behavioral effects of both protocols. Furthermore, a higher stimulation intensity was chosen for CB to account for the higher scalp to cortex distance^70^ and modelling work suggesting a maximum electric field strength in CB of about half the magnitude as in M1, when stimulated with the same current intensity^71^. The blinding procedures were carried out by a researcher (blinding assistant), not involved in other study-related assessments, data acquisition, or analysis. The randomization list was kept in a sealed envelope only accessible to study staff executing the stimulation protocols. Unblinding was done after all data was preprocessed and analyzed.

### Paired-pulse transcranial magnetic stimulation (ppTMS)

TMS was used to study short intracortical inhibition (SICI) and intracortical facilitation (ICF)^18,72^. The procedures are described in detail in our prior published work^24,73^. Monophasic pulses were delivered via two Magstim 200^2^ stimulators connected via a BiStim^2^ module and discharged through a figure-of-eight D70 alpha flat coil (Magstim Co Ltd, Whitland, United Kindom). The coil was placed over the motor hot spot for eliciting constantly the largest muscle responses in the first dorsal interosseous (FDI) muscle of the non-dominant, left hand. The coil was oriented that the handle pointed backwards with approximately a 45 degrees angle to midsagittal line. This resulted in a posterior-to-anterior induced currents in the underlying brain tissue. The coil position was kept in constant position for the further assessments. The conditioning pulses (CP) was adjusted to 80% of resting motor threshold (RMT)^74^ and the test pulses (TP) to an intensity that elicited motor evoked potentials (MEPs) of an ~ 1 mV peak-to-peak amplitude. TP and CP intensity were readjusted before each session to assess SICI and ICF in the stable range of their respective recruitment curves^18^. SICI was studied at an inter-stimulus-interval (ISI) of 3 ms at rest (SICI_rest_) and in premovement state (SICI_move_), see below. ICF was assessed at an ISI of 10 ms at rest (ICF_rest_). Eighteen trials were recorded per condition in a random order with inter-trail-jitter of 6 to 8 s for the rest assessments and in a pseudorandom order with inter-trial-jitter of 6–10 s for SICI_move_. Furthermore, SICI was tested in the premovement phase (SICI_move_) of a simple reaction task in the time zones around 20% and 90% of individual reaction time (RT)^73^. During the simple reaction time task, the participants were asked to perform left index finger abductions in response to a visual cue. The electromyography signal was sampled using disposable surface electrodes placed of the FDI in belly tendon montage via a 1902 amplifier (Cambridge Electronic Design Ltd, Milton, United Kingdom) at a sampling rate of 5 kHz and applying a 50 Hz to 1 kHz bandpass filter.

### Magnetic resonance imaging (MRI)

Multimodal MRI data were acquired with a 3 T Siemens Skyra MRI scanner (Siemens, Erlangen, Germany). T1-weighted anatomical images were acquired in coronal slices with the following parameters: repetition time = 2,500 ms, echo time = 2.12 ms, number of slices = 256, slice thickness = 0.94 mm, matrix size = 232 × 288, in-plane resolution = 0.83 mm × 0.83 mm, and flip angle = 9˚. Diffusion-weighted images consisted of 131 volumes were acquired in axial planes with the following parameters: b = 1500 s/mm^2^, repetition time = 10,000 ms, echo time = 82.00 ms, number of slices = 75, slice thickness = 2.00 mm, matrix size = 128 × 104, in-plane resolution = 2.00 mm × 2.00 mm, and flip angle = 90˚. The rs-fMRI gradient-echo EPI images consisted of 210 volumes and were acquired as axial planes with the following parameters: repetition time = 2,000 ms, echo time = 30.00 ms, number of slices = 32, slice thickness = 3.99 mm, matrix size = 72 × 72, in-plane resolution = 2.99 mm × 2.99 mm, and flip angle = 90˚.

### Data processing

Behavioural data of all 40 participants from all time points were acquired and entered the final analysis. Behavioural data were analysed with an in-house script scoring the correctly performed motor sequences averaged per block. Our primary outcome was the number of correctly performed sequences normalized to baseline. Training gain was quantified by the ratio of the last block of D2S2 and the first block of D1S1. Skill retention was determined via a retention index defined as the ratio of the average of the correctly performed sequences per block of a respective retention session normalized to the last training block in D2S2^13^. Temporal components of learning were operationalised by computing the differences (1) between the last block and the first block of a given training session for online learning and (2) between the first block of the subsequent session and the last block of the preceding session for offline learning^1,75^.

TMS data were acquired from 39 participants (in one participant no stable data could be obtained due to high thresholds). TMS data were analysed with an automated script implemented in Signal software (Cambridge Electronic Design Ltd, Milton, United Kingdom) quantifying the peak-to-peak MEP amplitude in a response window of TMS pulse plus 20 ms to 50 ms. All trials were visually inspected. Trial rejection criteria were: trials with documented failure of proper coil placement, muscle preactivation > 25 µV from baseline for rest and > 50 µV for event related trials in the time window of 100 ms before the TMS pulse, clear preactivation outside the critical window of 100 ms before the TMS pulse, no MEP defined as peak-to-peak amplitude < 0.05 mV for TP_only_ and ICF trials, overlap of the MEP with voluntary muscle contraction for the event-related trials. Amplitudes were averaged per condition and assessment time point. Magnitude of SICI and ICF was related to TP_only_ trials as follows:

$$\left(1\right) \quad SICI or ICF magnitude=\frac{mean(SICI or ICF trials)}{mean \left(T{P}_{only} trials\right) } \times 100$$

SICI_move_ modulation was expressed as follows (post: postD2 or postFU20, pre: preD1):

$$\left(2\right) \quad SIC{I}_{move} modulation=\frac{| SICI90{\%RT}_{post}-SICI20\%R{T}_{post}|}{| SICI90{\%RT}_{pre}-SICI20\%R{T}_{pre}|}$$

Data points of a given subject with less than 8 valid trials were excluded from further analysis (this case did not emerge for the SICI_rest_ and ICF_rest_ data, 12 out of 120 data points of the SICI_move_ data were excluded for the main reason of MEP overlap with the voluntary muscle activation).

MRI data were sampled from 35 participants (reasons for not acquiring MRI data were: N = 3 participants scheduling difficulties, N = 2 participants MRI contraindications). Diffusion-weight MRI data were analysed by means of MRtrix3 software (https://www.mrtrix.org/)^76^, FSL software package 5.0 (https://fsl.fmrib.ox.ac.uk/fsl/fslwiki/FSL) and FreeSurfer software package 6.0 (https://surfer.nmr.mgh.harvard.edu/). Cleaning of the images included: denoising, removal of Gibbs ringing artefacts, head motion and eddy currents correction. Brains were then skull stripped, fractional anisotropy (FA) maps were computed and registered to the Montreal Neurological Institute (MNI) standard space. Subsequently, the superior cerebellar peduncle (SCP) region was defined by using the Bayesian segmentation algorithm, based on a probabilistic atlas of the brainstem, available on FreeSurfer^77^ and registered to the MNI standard space. FA values were extracted within this region and their FA average was finally computed.

Rs-fMRI data were preprocessed using the tools in SPM12 (http://www.fil.ion.ucl.ac.uk/spm/) by following the order of spatial realignment for correcting for head movement, normalization into the same coordinate frame as the template brain in the MNI standard space, spatial smoothing with a Gaussian kernel of 8 mm full width at half maximum, linear detrending for removing systematic signal drift, regressing out the effects of head movement and non-neuronal fluctuations, and band-pass filtering at 0.01–0.08 Hz for removing physiological noise. From the preprocessed data, signals were extracted as the singular value decomposition of voxel-wise signals for the left cerebellum and the right M1. Functional connectivity between the left cerebellum and the right M1 was estimated by computing the correlation of signals and converting the correlation coefficient into a normally distributed value using the Fisher transformation.

### Statistical analysis

The statistical analysis was implemented in R (R Core Team, 2020)^78^. Linear mixed effects models were fitted using the *lmer()* function of the *lme4* package^79^. As random effects, we added intercepts for participants. To address RQ1-2, the respective active stimulation group of interest was compared to the sham group in an pairwise approach. For RQ3, the active stimulation group of interest multifocal M1-CB stimulation was compared to the conventional monofocal M1 stimulation group. The models were build up hierarchically using a multilevel approach starting from the null-model (intercept only model) and subsequently adding at first level CONDITION, at second level BLOCK (respectively SESSION), and at third level the CONDITION × BLOCK (respectively SESSION) interaction^80^. In the majority of cases, the residuals did not show obvious deviations from normality, defined as a skewness in-between -2 and 2^81^, in other cases (retention monofocal M1 vs. multifocal M1-CB, SICI_rest_, training gain, CV of training data) we performed a log-transformation of the dependent variable to meet this assumption. Statistical significance testing was done by applying likelihood ratio tests comparing the full model including the effect in question with the reduced model without the effect in question^82^. The cut-off for statistical significance was set at *p* < 0.05. For specific post hoc comparisons we conducted pairwise comparisons with least square means—*lsmeans()* function^83^—by applying a Tukey-correction for multiple comparisons. For the ppTMS data we performed an auxiliary analysis to assess for potential modulation from baseline by calculating one sample t-tests with Bonferroni correction. To assess for specific association of two variables of interest we calculated Spearman's rank correlations. Multiple linear regression analysis (*lm()* function) was used to predict the outcome variable training gain. Missing values (6.25% of cases) were imputed with median imputation. To allow direct comparisons of beta coefficients, the predictor variables were converted to z-scores. The final predictive model was determined by stepwise backward selection based on the Akaike information criterion (AIC). The selection process was implemented via the *stepAIC()* function^84^. The same approach was applied in an attempt to predict responsiveness for anodal M1 tDCS.

## Supplementary information

Supplementary Information

## Data Availability

The datasets generated during and/or analysed during the current study are available from the corresponding author on reasonable request.
