# Supplementary material for: Multifocal stimulation of the cerebro-cerebellar loop during the acquisition of a novel motor skill
Source: Sci Rep. 2021 Jan 19;11:1756. doi: 10.1038/s41598-021-81154-2 (PMC7815761; doi:10.1038/s41598-021-81154-2)
Supplement: Supplementary file 1 — Supplementary Information [file 41598_2021_81154_MOESM1_ESM.pdf]

Supplementary Information: Multifocal stimulation of the cerebro-cerebellar loop during the acquisition of a novel motor skill

Maximilian J. Wessel[1,2,*], Chang-hyun Park[1,2], Elena Beanato[1,2], Estelle A. Cuttaz[1,2], Jan E. Timmermann[3], Robert Schulz[3], Takuya Morishita[1,2], Philipp J. Koch[1,2] & Friedhelm C. Hummel[1,2,4]

[1] Defitech Chair of Clinical Neuroengineering,
Center for Neuroprosthetics (CNP) and Brain Mind Institute (BMI),
Swiss Federal Institute of Technology (EPFL), Geneva, Switzerland
[2] Defitech Chair of Clinical Neuroengineering,
Clinique Romande de Réadaptation,
Center for Neuroprosthetics (CNP) and Brain Mind Institute (BMI),
Swiss Federal Institute of Technology (EPFL Valais), Sion, Switzerland
[3] Department of Neurology, University Medical Center Hamburg-Eppendorf, Hamburg, Germany
[4] Clinical Neuroscience, University of Geneva Medical School, Geneva, Switzerland

*Correspondence:
Dr. Maximilian J. Wessel
Defitech Chair of Clinical Neuroengineering
Center for Neuroprosthetics (CNP) and Brain Mind Institute (BMI)
Swiss Federal Institute of Technology (EPFL)
Campus Biotech,
Chemin des Mines 9
1202 Geneva
email: maximilian.wessel@epfl.ch
Tel: +41 21 695 50 90

Analysis of task performance (pseudorandom blocks)

Research question	M±SEM (chronological order)	CONDITION	SESSION	CONDITION x SESSION	Post-hocs
RQ1 (M1vsSham)	M1: 8.70±0.62, 11.80±1.43, 11.60±0.73, 10.10±1.03, 13.40±1.00 Sham: 11.40±1.42, 13.50±1.48, 14.70±2.38, 12.30±1.44, 15.70±2.16	$\chi^2(1)=2.00$, $p=0.16$	$\chi^2(4)=28.31$, $p<0.001$*	$\chi^2(4)=0.78$, $p=0.94$	BvsR1: p=0.029* BvsR2: p=0.0053* BvsR3: p=0.68 BvsR4: p<0.001* R1vsR2: p=0.98 R1vsR3: p=0.46 R1vsR4: p=0.19 R2vsR3: p=0.17 R2vsR4: p=0.49 R3vsR4: p=0.0020*
RQ2 (CBvsSham)	CB: 11.10±2.37, 13.90±3.33, 14.10±3.17, 11.70±2.05, 15.40±3.17 Sham: 11.40±1.42, 13.50±1.48, 14.70±2.38, 12.30±1.44, 15.70±2.16	$\chi^2(1)=0.01$, $p=0.92$	$\chi^2(4)=19.58$, $p<0.001$*	$\chi^2(4)=0.30$, $p=0.99$	BvsR1: p=0.17 BvsR2: p=0.036* BvsR3: p=0.96 BvsR4: p=0.0013* R1vsR2: p=0.97 R1vsR3: p=0.52 R1vsR4: p=0.43 R2vsR3: p=0.18 R2vsR4: p=0.82 R3vsR4: p=0.013*
RQ3 (M1-CBvsM1)	M1-CB: 10.40±1.36, 14.30±2.09, 12.20±1.30, 13.40±1.81, 17.20±2.18 M1: 8.70±0.62, 11.80±1.43, 11.60±0.73, 10.10±1.03, 13.40±1.00	$\chi^2(1)=1.84$, $p=0.18$	$\chi^2(4)=46.37$, $p<0.001$*	$\chi^2(4)=6.03$, $p=0.20$	BvsR1: p<0.001* BvsR2: p=0.024* BvsR3: p=0.041* BvsR4: p<0.001* R1vsR2: p=0.57 R1vsR3: p=0.44 R1vsR4: p=0.034* R2vsR3: p=1.00 R2vsR4: p<0.001* R3vsR4: p<0.001*

Supplementary Table S1. Effects of practice but not stimulation on task performance. Task baseline and blocks with pseudorandom, untrained sequence served as performance probes. Analysis was done with raw non-baseline related data. M1, monofocal M1 stimulation group; Sham, sham group; CB, monofocal cerebellar stimulation group; M1-CB, multifocal motorcortico-cerebellar stimulation group; M, mean; SEM, standard error of the mean, B, Baseline; R1, block with pseudorandom sequence D1S1; R2, block with pseudorandom sequence D1S2; R3, block with pseudorandom sequence D2S1; R4, block with pseudorandom sequence D2S2. * depicts $p<0.05$.

Applied sequences in the sequential finger tapping task (SFTT)

Block	Sequence
Baseline	3-5-4-3-4-2-5-2-4
Training	4-2-3-5-3-4-2-5-3
Pseudorandom D1S1	5-4-2-3-5-2-3-4-3
Pseudorandom D1S2	2-4-3-5-3-2-5-3-5
Pseudorandom D2S1	3-2-4-5-2-3-5-4-5
Pseudorandom D2S2	4-5-4-2-3-2-5-4-2

Supplementary Table S2. Applied sequences in the sequential finger tapping task (SFTT). 2 = index finger, 3 = middle finger, 4 = ring finger, 5 = little finger. D1S1, day 1 session 1; D1S2, day 1 session 2; D2S1, day 2 session 1; D2S2, day 2 session 2.

Power simulation for increasing the sample size

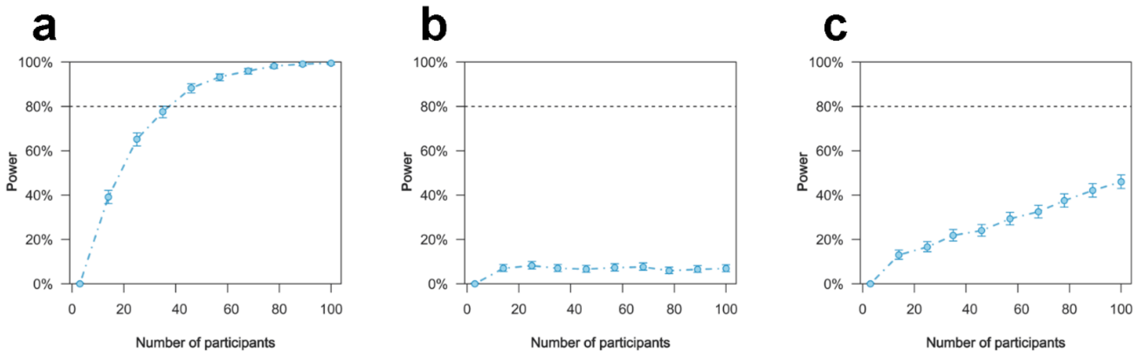

Supplementary Figure S1. Simulated power curves to inform future replication studies for the RQs 1-3 studying the effect of the applied tDCS protocols on motor training. The simulations for increasing the number of participants to N = 100 were implemented with the R package simr and based on Monte Carlo simulations[1]. The simulations were based on the obtained estimates for the fixed effect CONDITION. (a) RQ1 - additive effect of multisession monofocal M1 stimulation, (b) RQ2 - multisession cerebellar stimulation, (c) RQ3 - sequential multifocal motorcortico-cerebellar stimulation. Dotted line depicts the traditionally desired power level of 80%. For the RQ2 and 3, for which the applied tDCS protocols did not indicated a significant stimulation (CONDITION) effect, the simulation indicated that a sample size increase to N = 100 would not critically increase the level of power (see panel b and c). Margins of error depict 95% confidence intervals.

References:

1. Green, P. & MacLeod, C. J. SIMR: an R package for power analysis of generalized linear mixed models by simulation. *Methods Ecol. Evol.* **7**, 493–498 (2016).
